# Designing, Conducting, and Documenting Human Nutrition Plant-Derived Intervention Trials

**DOI:** 10.3389/fnut.2021.782703

**Published:** 2021-12-23

**Authors:** Connie M. Weaver, J. Kalina Hodges

**Affiliations:** ^1^Department of Nutrition Science, Purdue University, West Lafayette, IN, United States; ^2^Department of Human Sciences, The Ohio State University, Columbus, OH, United States

**Keywords:** best practices, human nutrition, clinical trials, plant-derived interventions, study design

## Abstract

Best practices for designing, conducting, documenting, and reporting human nutrition randomized controlled trials were developed and published in Advances in Nutrition. Through an example of the randomized clinical trial on blueberries and bone health funded by the National Institutes of Health, this paper will illustrate the elements of those best practices that apply specifically to plant-based intervention clinical trials. Unique study design considerations for human feeding interventions with bioactive plant compounds include the difficulty of blinding the intervention, background nutritional status of participants, carry-over effects of the intervention, benefits of a run-in period, lack of safety/tolerability data, and nutrition-specific regulatory policies. Human nutrition randomized controlled trials are the gold standard for establishing causal relations between an intervention and health outcome measures. Rigorous studies and documentation define the quality of the evidence-base to inform public health guidelines and to establish personalized dietary recommendations for the health-promoting plant components.

## Introduction

A comprehensive resource on best practices for designing, conducting, documenting, and reporting human nutrition randomized controlled trials (RCT) for everyone involved in the clinical trials research enterprise was published as a series of manuscripts in the American Society for Nutrition journal, Advances in Nutrition (1–5). A two-part training workshop on these articles was offered by the American Society for Nutrition in July 2021 with plans for future repeat offerings of the workshop. The National Institutes of Health (NIH) Clinical Trials Award (CTSA) program and the American Society for Nutrition appointed members of a working group called Nutrition InteRvention ReSearcH (NURISH) to develop best practices and train researchers, institutional representatives, research sponsors, and regulators to improve rigor of human nutrition research that provides the evidence-base for making policy decisions regarding diet with the ultimate goal of improving human health.

The articles that described best practices for human nutrition RCTs cover general considerations unique to nutrition interventions such as the difficulty of blinding the intervention, baseline nutritional status of participants, carry-over effects, run-in periods, and safety of the intervention. The aim of this perspective is to discuss these best practices in the context of human plant-derived interventions. Key concepts are illustrated with examples from an NIH-funded RCT of the dose-response effects of freeze-dried blueberry powder on bone calcium retention in postmenopausal women (NIH/NICCH grant: R01 AT008754; ClinicalTrials.gov ID: NCT02630797). This RCT employed novel bone labeling technology and was conducted in healthy participants, thus, results are more generalizable compared with trials in patient populations. The trial required careful monitoring of participant well-being to avoid attrition and presented many considerations relevant to the design and conduct of plant-derived interventions. Although our primary outcome of net bone calcium retention in postmenopausal women is a specific outcome, the methodologies described herein are relevant to other aging and chronic disease related outcomes including cardiovascular, cardiometabolic, cognitive, inflammatory, and gastrointestinal outcomes.

## Design of Randomized Controlled Trials

Identifying a research question that is important, novel, and feasible to address with the available methods informs the study aims, hypotheses, design, and procedures. RCTs are considered to provide the most reliable evidence on the effectiveness of interventions, because they minimize the risk of confounding by other factors, and thus, help to establish causal relations between the exposure and health outcome measures.

Plant-derived interventions have unique challenges. For example, bioactive components such as polyphenols are not under the same homeostatic control mechanisms as nutrients. Bioavailability studies of these bioactive compounds show that absorption is limited, but we know little about their metabolism, distribution, and excretion. They undergo extensive metabolism by the gut microbiota, which complicates the causal pathway. Their concentration in plasma is usually low and they may be unstable; thus, their quantification requires highly sensitive and selective analytical techniques (i.e., high-performance liquid chromatography and mass spectrometry).

**Figure 1:**
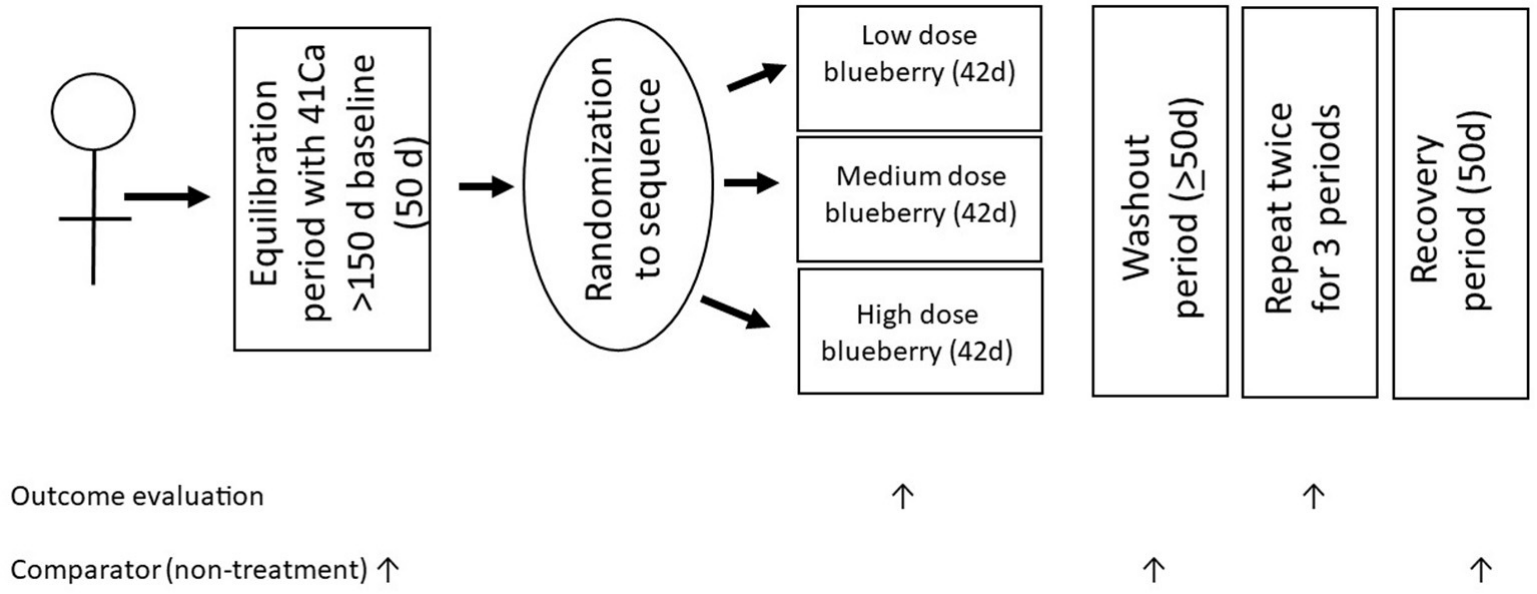
Randomized crossover study design for blueberries and bone RCT.

The characteristics of the example study on blueberries and bone are presented in the format of the CONsolidated Standards Of Reporting Trials (CONSORT) guidelines (6) (Table 1), which were published in 2010 with the aim of improving quality of clinical trial reports. The blueberry and bone RCT used the gold standard of a randomized, crossover trial design as illustrated in Figure 1 and investigated changes in net bone calcium retention (a measure of bone loss and our primary outcome) in response to a dietary intervention with freeze-dried blueberry powder. Changes in net bone calcium retention were quantified with the use of a rare, long-lived radiotracer, ^41^Ca, measured in urine by accelerator mass spectrometry (AMS) (7). The use of this ultra-sensitive measurement method and the ability to make within-subject comparison of treatment vs. control over time greatly increased the power to assess the efficacy of our intervention, thereby decreasing sample size and intervention duration compared with trials using the traditional bone density measurement approach. The AMS method also allows for a relatively more rapid screening of several interventions than is feasible with bone mineral density or fracture outcomes. The equilibration period, which is necessary to allow bone to be labeled with the rare isotope, also serves as a run-in period to determine participant commitment to the protocol. In the blueberry and bone RCT described in Table 1 and Figure 1, we tested three doses of blueberry powder in 13 participants over 1.5 years compared to a typical parallel arm study in two groups of >60 each requiring up to 4 years to establish intervention-related changes in bone mineral density using densitometry. The multiple studies conducted to validate our study design were described by Weaver et al. (7).

**Table 1:** CONSORT guidelines applied to Blueberries and Bone randomized controlled trial.

**Section/topic**	**Guideline**
Title	Dose-response effect of blueberries on net bone calcium retention in postmenopausal women: a randomized controlled trial
Abstract	Structured summary of trial design, methods, results and conclusions
Introduction	
Background	Preclinical studies have shown a benefit of blueberry consumption on bones.
Objective	To evaluate the dose response effects of blueberry consumption on bone calcium retention in humans
Hypothesis	Increasing dose of freeze dried whole blueberry powder will decrease ^41^Ca excretion from bone in postmenopausal women.
Methods	
Trial design	Randomized, crossover; changes to trial design: intervention periods reduced from 50 to 42 d
Participants	Healthy postmenopausal women (>4 years postmenopause) aged 45–70 years, not on osteoporosis treatment medication or other medicines that influence bone loss for >6 months prior to study initiation, not osteopenic or with a history of bone fractures, and willing to discontinue self-selected natural products
Study settings	Free living with clinical visits at the University Clinical Research Center
Intervention	Three doses of freeze-dried whole blueberry powder, i.e., low (17.5 g equivalent to 0.75 cup fresh berries), medium (35 g equivalent to 1.5 cups fresh berries), and high (70 g equivalent to 3 cups fresh berries)
Outcomes	The primary outcome measure was urinary ^41^Ca excretion from pre-labeled bone (equilibrated for >150 days post dose for soft tissue ^41^Ca to be excreted) expressed as % net bone Ca retention compared to non-treatment periods of baseline and washout. Secondary outcomes included diet analysis, polyphenolic content of blood and urine, and bone turnover biomarkers.
Changes to outcomes	None
Sample size	Eighteen participants were enrolled, 16 initiated the study, and 13 completed the entire study giving us 80% power to detect a 0.9% improvement in ^41^Ca retention based on effect size and retention in previous similar studies conducted by our research group.
Interim analyses and stopping guidelines	Our *a priori* rules were to stop the intervention if adverse events or new information raised safety concerns or if recruitment failed.
Randomization	The dose sequence was generated by a random generator program by the study statistician. The products were coded according to dose by the Clinical Research Center kitchen staff. The Study Coordinator recruited participants, managed the clinical visits, and supervised sample preparation for analysis.
Blinding	Products were prepared by kitchen staff to vary only the dose of blueberries. Research staff and participants were blind to the product codes according to dose.
Results	
Participant flow	Of the 16 participants who enrolled and began the study, 13 completed the entire study and constituted the sample for analysis.
Losses and exclusions	Seventeen were found ineligible on screening, three dropped out before the first intervention, and one completed two of the three phases before she moved out of the area.
Reason for stopping trial	The IRB suspended the trial for 2 months because of an adverse event.
Baseline data	Baseline and clinical characteristics were collected for the participants.
Outcomes	Will be reported elsewhere.
Discussion	Limitations and interpretation will be reported elsewhere.
Generalizability	This was an efficacy, not an effectiveness trial, in a small group of reasonably similar postmenopausal women.

A limitation of the crossover design is a potential carry-over effect from one intervention period to the next. In our previous studies of bone health, only the intervention with bisphosphonates (osteoporosis treatment drug that is retained in the skeleton) precluded urinary ^41^Ca:Ca ratios from returning to baseline after a 50-day washout period (8). Thereafter, positive control long-acting drugs were given as the last intervention rather than in a randomized order. Our protocol is most feasible for small efficacy studies with limited generalizability compared to effectiveness studies. Effectiveness studies in a “real world setting with a more generalized population” typically follow an efficacy study before policy is developed.

## Participants

General participant considerations were discussed in Lichtenstein et al. (2). The choice of study population influences the generalizability of the study results. The more diverse the population, the more generalizable the results, although responses to a plant-derived intervention are likely to vary even in a relatively uniform sample due to mediating factors, which are difficult to control even in an RCT (e.g., background diet, physical activity, gut microbiota). The likelihood of high variability requires a larger sample size, which imposes a higher cost of the study. Patients with a specific disorder of interest may be more responsive to the intervention than a generally healthy population. However, recruitment of patients requires careful screening of medical histories to exclude participants with medications or conditions potentially confounding to the outcome of interest. Participants in our blueberry and bone RCT were healthy women stable to menopause. Postmenopausal women are most vulnerable to bone loss, and therefore, most likely to benefit from dietary interventions that would ameliorate bone loss due to estrogen deficiency. We selected women at least 4 years menopausal because rapid and inconsistent bone loss during the perimenopausal period would shift urinary ^41^Ca:Ca ratios independent of diet effects. To control fluctuation of two nutrients known to influence bone loss, we provided calcium and vitamin D supplements throughout the study. We also monitored serum 25(OH)D to ensure that status did not change throughout the study. Vitamin D status can also affect immune function, and thus indirectly the health outcome. Sex differences were not determined in our RCT, but preclinical studies of blueberries showed important sex differences of bone in response to blueberry feeding (9). Determining sex differences should be part of the study design for plant-derived interventions whenever possible.

## Intervention, Background Diet, Randomization, and Adherence

A plant-derived intervention can be provided in the form of a food, an ingredient, a supplement, or an extract. When selecting the intervention, form and dose level, safety, acceptability, and practicality are key considerations. Testing several doses within the safety limit is useful when the dose-response relationship is not established, as is the case for many plant bioactives. Single vs. repeated dosing can also alter bioavailability and pharmacokinetic response to polyphenols (10).

In our blueberry and bone RCT, the intervention consisted of three dose levels (low: 17.5 g/d, medium: 35 g/d, and high: 70 g/d) of freeze-dried whole blueberry powder incorporated into three products: a drink, a spread, and granola bites (cubic bars) consumed as part of a self-selected diet. Considerable product development efforts were undertaken to formulate products that did not require heat for preparation (high temperatures may degrade certain bioactive constituents), were palatable at the provided doses of blueberry powder (equivalent to 0, 1.5, and 3 cups of whole blueberries), and practical for consumer use with minimal preparation and storage requirements. Importantly, we also verified stability of the polyphenol profile in the freeze-dried blueberry powder and the intervention products throughout the study. The concentration of total polyphenols in freeze-dried powder was 35.2 ± 0.6 mg/g, which was consistent with data provided by the manufacturer. The concentration of total polyphenols in the intervention products ranged from 522 to 613 mg per each low-dose serving indicating that processing lowered the concentration by <20%. Participants taste tested all products prior to study initiation and selected two products to consume daily, one in the morning and one in the evening. Our choice of a self-selected diet is more generalizable than the use of a controlled diet. Moreover, when we compared an intervention of a prebiotic as part of a controlled diet (11) vs. a self-selected diet (12) on calcium absorption efficiency in a cross-over study in adolescents, results did not differ. However, the self-selected diet could introduce potential confounding effects of nutrient and bioactive compound intakes that blunt the effect of the intervention.

Formulating a comparator control or placebo can be challenging in plant-derived human intervention trials, especially if the intervention is rich in pigment, as with blueberries. Both participants and researchers may be able to infer treatment and/or dose by color if the products are compared side by side. The choice of comparator product or placebo is further complicated by the potential interaction of plant-derived compounds with other product components (e.g., proteins) or the interaction of placebo components (e.g., fibers) with gut microbiota. In a prior trial of prebiotic fibers (11), we have used maltodextrin as the comparator, but there is some concern that maltodextrin alters the gut microbiome, which acts as the mediator for health outcomes.

In our blueberry and bone RCT, double blinding was implemented by coding of products by research kitchen staff prior to dispensing them to the study coordinator who delivered them to participants. Minimally, single blinding can usually be accomplished if sample analysis is performed by researchers blinded to the intervention.

In crossover trials, the use of comparator or placebo may be substituted by adding a control period, during which participants undergo the same procedures as during the experimental period without consuming the intervention product. In our blueberry and bone RCT, multiple untreated periods (baseline, washout, and recovery periods) served as the control periods. We opted not to use a placebo based on our previous RCT of hesperidin using the same protocol, which demonstrated that results of the placebo period were indistinguishable from those of the untreated periods (13). Eliminating the placebo also reduced participant burden by shortening the study by one placebo period and the subsequent washout (~100 days).

The randomized schedule for the sequence of interventions was provided by our statistician. More complex studies that assign participants to different groups may randomize by blocks or clusters to minimize bias, ensure that groups have similar baseline characteristics, or minimize contamination of the intervention as discussed in Lichtenstein et al. (2).

A common approach to monitor adherence and limit confounding by other nutrients/dietary bioactives is to provide participants with a list of polyphenol-rich foods to avoid and/or limit. In the blueberry and bone RCT, we instructed participants to limit the consumption of polyphenol-rich foods and collected diet records to quantify polyphenol intake. In addition to the self-reported diet records, the study coordinator kept a record of returned uneaten foods (<2% returned) and spot and 24-h urine samples were analyzed for polyphenols. To minimize attrition, e-mail reminders and calendars with all study visits were sent to participants on a weekly basis. A run-in period is also useful to determine participant commitment to the study protocol, but it may alter baseline measures (2).

## Outcome Measures

When selecting outcome measures, biomarkers for outcomes of interest should be on the causal pathway and validated for predicting the end condition. In the blueberry and bone RCT, changes in the primary outcome, i.e., urinary ^41^Ca:Ca, have been validated against changes in bone mineral density (14). Bone mineral density is also a biomarker for fracture risk, the health outcome of interest, and is one of several biomarkers approved by the FDA. Other FDA approved qualified biomarkers for specific chronic diseases include serum cholesterol and blood pressure for cardiovascular disease, adenomatous colon polyps for colon cancer, and elevated blood glucose and insulin resistance for diabetes (15). An active area of research is identification of validated biomarkers of exposure and predictors of health outcomes. In nutrition intervention studies, the exposure is often estimated by self-reported dietary assessment methods. Best practices for this approach have been reported (16), but more objective approaches are desirable. Davy and Davy (17) make a strong case for controlled feeding studies to reduce variability of exposure. Genetics, metabolomics, and microbiome profiles are some of the approaches being investigated to identify good biomarkers of both exposure and outcomes and to account for potential confounders. In our trial, we measured phylogenetic diversity of bacterial communities using 16S rRNA sequencing. The statistical analyses of microbial taxa, alpha and beta diversity, and correlations with polyphenol metabolites and bone health outcomes are ongoing.

Few studies using plant-derived interventions consider the timing of sample collection relative to the ingestion of intervention for monitoring exposure. Most studies default to collecting fasting urine and/or blood, although serum concentrations of plant-derived bioactives tend to be more variable than in urine. Because the half-life of bioactives is usually short (<12 h), a fasting sample could miss their appearance if consumed the day before. Furthermore, there is more natural variation in urinary excretion of bioactives than para-amino benzoic acid, which frequently serves as a marker of urine collection completeness due to its 100% excretion in urine. In our blueberry and bone RCT, we used 24-h urine samples for both the primary outcome and polyphenol outcomes, as well as for monitoring adherence. The most abundant metabolites recovered from 24-h urine included anthocyanin metabolite delphinidin-3-glucuronide and two phenolic acids, hippuric acid and caffeic acid sulfate. Continuing efforts are focused on estimating the interindividual variation in blueberry polyphenol metabolite excretion as well as the changes due dosage.

Timing of ingestion of the bioactives can influence not only adherence measures but also the outcome measures directly or indirectly by altering mechanisms that influence health outcomes. A study of the effect of morning vs. evening consumption of chocolate showed that, compared to evening consumption, morning consumption decreased *ad libidum* energy consumption, fasting glucose, and waist circumference, increased lipid oxidation, sleep onset variability and temperature rhythms, and altered microbiota composition and function (18).

## Documentation and Regulation

The importance of documentation and meeting regulatory requirements for human nutrition RCTs was described in detail by Weaver et al. (3). A plant-derived intervention may require pre-approval by the Food and Drug Administration (FDA), even if it is a commonly consumed food ingredient or product. For our trial, we assessed multiple varieties of wild and cultivated blueberries using a principal component analysis and selected 6 with the most divergent phenolic profiles. These varieties were then tested for polyphenol bioavailability in a preceding animal study. The material of choice for the human trial was a composite of several low-bush varieties (*Vaccinium angustifolium*) sourced from a number of growing regions including Quebec, Newfoundland, Maine, and Nova Scotia. The composite was prepared by Wild Blueberry Association of North America and freeze-dried by FutureCeuticals, Momence, IL. The powder was packed in multilaminate foil pouches and stored at 4°C. The powder was accompanied by a certificate of analysis to ensure that it passed the safety test, and is also available commercially.

Prior to initiating our blueberry and bone RCT, we obtained a waiver decision by the FDA that an Investigative New Drug (IND) application was not required for trial initiation. An IND is required if the RCT is evaluating diagnosis, cure, mitigation, treatment, or prevention of a disease. This can be off-putting to a commercial supplier of a product who does not wish to have a public record that the FDA is evaluating their product as a drug when they are marketing it as a dietary supplement.

Aside from FDA, the sponsor, safety, and ethical committees may require certain characteristics of the test substance to be reported prior to trial initiation. These may include absorption, distribution, metabolism, and excretion determined in preclinical studies. In our RCT, the funding agency required an analysis of the blueberry polyphenolics, the hypothesized bioactives.

Ensuring participant safety and data integrity in a plant-derived intervention trial may require the oversight of multiple ethics and regulatory committees. An Institutional Review Board (IRB) at the research institution or a commercially contracted IRB reviews the study application. A Data Safety and Monitoring Board (DSMB) or Investigational Monitoring Committee (IMC) may be appointed by the funding agency or the Principal Investigator. The DSMB committee is RCT-specific and its members must have scientific expertise in the topic of investigation and experience conducting similar studies. For our blueberry and bone RCT, an IMC and Data Safety and Monitoring Plan (DSMP) (Table 2) were required. The DSMP included safety of the ^41^Ca method, data protection, integrity, and confidentiality. Although consumption of freeze-dried blueberry powder was not expected to have any adverse effects, a standard process for reporting any adverse events was also included.

**Table 2:** Data safety and monitoring plan for blueberries and bone RCT.

**I. Study identification number**
**A. NIH/NCCIH study number:** R01 AT008754; ClinicalTrials.gov ID: NCT02630797
**B. Study title:** Blue Berries and Bone
**C.** ***Name of Principal Investigator (PI):*** Connie Weaver, PhD
**D.** ***Name and role of Co-***Is: Gorge McCabe, PhD-statistician; Munro Peacock, MD-study physician
**II. Study overview**
**A. Brief description of the purpose of the Study:** The overall goal of this study is to evaluate the dose response effects of continuous blueberry consumption over a 50 day period on net bone calcium retention in healthy-post menopausal women.
**B. Adherence statement:** The Data Safety and Monitoring Plan (DSMP) outlined below for RO1 AT0087541 will adhere to the protocol approved by the Indiana Clinical and Translational Science Institute (CTSI) Research Review Committee and the Purdue University Institutional Review Board (IRB).
**III. Confidentiality**
**A. Protection of subject privacy**
During the study, all records associated with each person's participation in the study will be managed using the usual confidentiality standards applicable to medical records. All of the materials collected are for research purposes only, and data will be kept in strict confidence. No information will be given to anyone without permission from the subject. The consent form includes the informed consent statements required by Purdue University. Confidentiality will be ensured by use of identification codes. All data, whether generated in the laboratory or at a clinical visit, will be identified with a randomly generated identification code unique to the subject.
**B. Database protection**
The database will be secured with password protection. Electronic communication with outside collaborators will involve only unidentifiable information. All paper source documents from all enrolled participants, including lab reports and subject study binders, will be stored in a locked cabinet in a locked storage facility, which is only available to the study staff. Electronic data will be stored in a password protected account.
**C. Confidentiality during Adverse Event (AE) reporting**
AE reports and annual summaries will not include subject or group identifiable material. Each report will only include the identification code.
**IV. Adverse event information**
**A. Definition**
An adverse event (AE) is any untoward medical occurrence in a subject during participation in the clinical study. An adverse finding can include a sign, symptom, abnormal assessment including laboratory test value, vital signs or any combination of these.
A serious adverse event (SAE) is any AE that results in one or more of the following outcomes:
• Death
• A life-threatening event
• Inpatient hospitalization or prolongation of existing hospitalization
• A persistent or significant disability/incapacity
• A congenital anomaly or birth defect
• An important medical event based upon appropriate medical judgment
**B. Classification of AE Severity**
AE's will be labeled according to severity, which is based on their impact on the subject. An Ae will be termed “mild” if it does not have a major impact on the subject, “moderate” if it causes the subject some minor inconvenience, and “severe” if it causes a substantial disruption to the subject's well-being.
**C. AE attribution scale**
AE's will be categorized according to the likelihood that they are related to the study intervention. Specifically they will be labeled definitely unrelated, definitely related, probably related, or possible related to the study intervention.
**D. Expected risks**
Expected risks to the subject are as follows:
• Radioisotope dose: the lifelong radiation exposure associate with receiving Ca-41 is <1/100,000th of a set of dental x-rays.
• Blood collection: The health risks involved in this study include drawing blood which can lead to bruising and infection. Precautions will be taken to minimize this risk by using sterile technique and applying pressure to the site after the needle is withdrawn. Professional trained staff will be present at all study visits at the Purdue University site to ensure necessary interventions in the event of adverse events. Trained staff at Indiana University School of Medicine (IUSM) will administer the ^41^Ca.
• Dual energy x-ray absorptiometry: The average absorbed dose of radiation from the bone measurement is 1.424 mRem. In comparison the average exposure from a set of dental x-rays is 1 mRem and from a chest x-ray is 6 mRem.
• We know of no risks associated with consumption of blueberries. However, project personnel will contact each subject at least once during the intervention phases to inquire about such events. The occurrence of adverse events will also be queried during each clinical visit.
**E. AE reporting and follow-up**
Adverse Event Report Forms are to be completed at each clinical visit.
Individual data will be summarized and reported every 6 months to the Data Safety and Monitoring Committee (DSMC), IRB and other oversight organizations when necessary.
**F. SAE reporting**
SAEs that are unanticipated, serious, and possibly related to the study intervention will be reported to the DSMC, IRB, Indiana CTSI, FDA, and NCCIH in accordance with requirements.
• Unexpected fatal or life-threatening AEs related to the intervention will be reported to the NCCIH Program Officer within 7 days. Other serious and unexpected AE's related to the intervention will be reported to the NCCIH Program Official within 15 days.
• Anticipated or unrelated SAEs will be handled in a less urgent manner but will be reported to the DSMC, Indiana CTSI, NCCIH, and other oversight organization in accordance with their requirements. In the annual AE summary, the DSMC Report will state that they have reviewed all AE reports.
**V. Data quality and safety review plan and monitoring**
**A. Data quality and management**
**1. Description of plan for data quality and management**
The study staff will review all data collection forms on an ongoing basis for data completeness and accuracy as well as protocol compliance. Someone other than the study staff will enter data into the password protected spread sheets. A summary of the data review will be reported to the DSMC.
**2. Frequency of data review**
Data will be reviewed by the PI and/or Study Director every 6 months.
**B. Subject accrual and compliance**
**1. Recruitment of subjects and compliance with inclusion/exclusion criteria**
During the initial recruitment period the PI will review rate of enrollment and compliance with inclusion and exclusion criteria monthly until enrollment goals are met.
**2. Reporting of compliance to intervention**
Products to be consumed will be delivered bi-weekly to the participants. Any products that have not been consumed will be returned and the numbers will be recorded on an appropriate spread sheet. Participants will be provided with a calendar that is designed to report the date and time of consumption of the products. The PI and Director will review these records monthly and report to the DSMC if compliance falls below 50%.
C. Justification of sample size
We will use the same ^41^Ca methodology that we have used in several other studies to evaluate the effects of interventions on net calcium retention. The response variable is the log of the ratio of ^41^Ca to total Ca in urine samples. Specifically, for each subject a simple linear regression is constructed using all control and recovery periods. This line is then used to estimate values for the treatment periods. Differences between the estimates using the control data and the actual treatment values are averaged for each subject and then combined across subjects. Results are back transformed to obtain estimates of net calcium retention for each treatment. The crossover design is particularly efficient for these studies because the same control information can be used for each of the treatments. Based on our previous data, we will have 80% power to detect a 0.9% improvement in net calcium retention with 13 subjects.
**D. Stopping rules**
This study will be stopped prior to its completion if: (1) the intervention is associated with adverse effects that call into question the safety of the intervention, (2) difficulty in recruitment or retention that may impact appropriate evaluation of endpoints, (3) any new information becomes available during the trial that necessitates stopping the trial.
**E. Designation of a monitoring committee**
The PI will designate a DSMC to perform a review of ongoing study progress and safety. The members will not be associated with this research project.
**F. Safety review plan**
Study progress and safety will be reviewed quarterly. Progress reports will be provided to the DSMC. A summary of details of subject recruitment, retention and AE's will be included. An annual report will include evaluation of recruitment and retention as well as continuation of the study.
**G. Study report outline**
The study team will develop a plan for writing a study report that will include the following topics:
Study status including issues or problems, a study description including projected timetable, recruitment status, enrollment data, as well as summary of AE's and safety assessment.
**VI. Informed consent**
Written informed consent will be obtained from each participant before the screening process. A member of the study team will summarize the procedures involved in the study and answer any questions that the subject might have.
The participant will acknowledge their willingness to participate in the study by signing the consent form in the presence of the study staff member.
**VII. Reporting changes in study status**
Any disruption in the study status as a result of decisions made by FDA, IRB, or one of the study investigators will be reported to the funding agency (NICCH) within one business day.

During the trial, a participant reported that the blueberry drink irritated a mouth sore, which was resolved by diluting the drink. The IRB suspended the study until we modified the consent form to include a statement that consumption of products with blueberry powder may cause oral irritation and re-consented all participants. The IRB also requested input from the IMC, which subsequently reported that it considered oral irritation to be a minor event and recommended that the study be permitted to resume. The suspension caused extra participant burden for those who were in the middle of an intervention when the study was suspended. Permission to repeat or extend a phase also required IRB approval.

The reporting of the suspension of the RCT by the IRB to the funding agency prompted an external audit by the sponsor. The external audit was conducted over 3 days and involved a review of regulatory documents, consent forms, source documentation, intervention preparation and dispensing records, study data, and a summary meeting with the Principal Investigator and the study staff. The same review standards and assessment criteria were applied as are used in monitoring pharmaceutical trials of substances with unknown and potentially serious side effects. The monitoring visit culminated with a report stating that no corrective action was necessary; however, 13 recommendations were provided for creating additional documents to track compliance with regulations and data integrity, e.g., delegation of authority log, concomitant medication use log, specimen tracking log, and internal quality assurance log. Assembling and maintaining such level of documentation may be a challenge in human nutrition trials, which do not usually have the support of multiple clinical research associates and data managers, but is nevertheless recommended by external auditing agencies.

## Standard Operating Procedures

**Figure 2:**
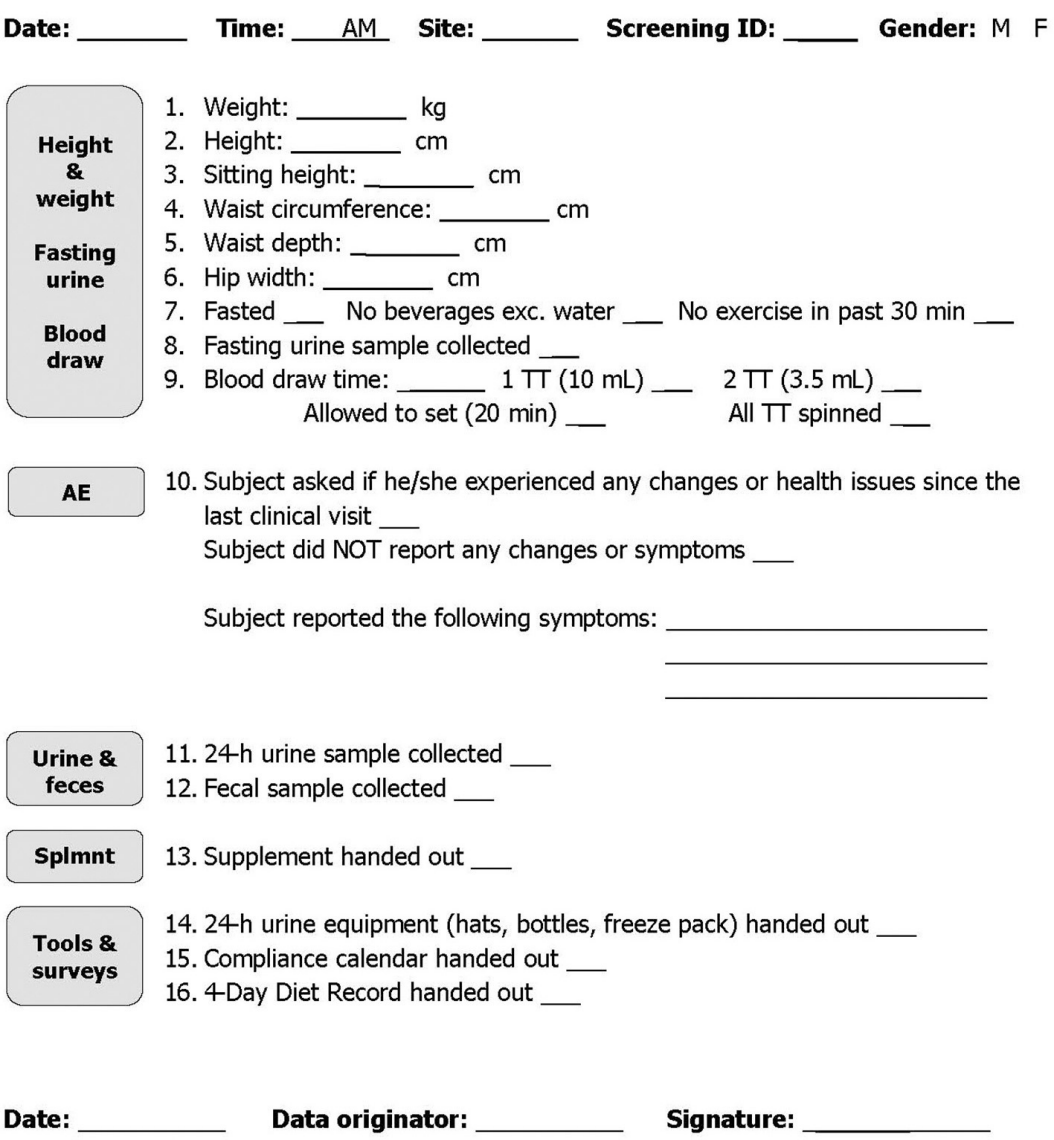
Berries and bone treatment week 6 flowsheet.

Specific procedures need to be established to estimate and monitor exposures, adherence, safety, and efficacy of the intervention. A standard operating procedure (SOP) for each should be in place prior to trial initiation to assure data quality and improve reproducibility, especially if the trial is a multi-center study. In the blueberry and bone RCT, we used flowsheets outlining the steps of each participant visit (Figure 2) to standardize data collection procedures and monitor participant safety during the study. We also developed an SOP of good documentation practice (Table 3) to ensure that all study aspects are properly recorded.

**Table 3:** Weaver laboratory good documentation practice standard operating procedures.

1.	Original source documents regarding study procedures and subject health (questionnaires, flowsheets, screening lab results, etc.) will be reviewed and filed before subjects move from one phase of a study to another. These documents will be retained after data entry for all studies and stored securely in a locked cabinet.
2.	The source of data (whether by self-report or by data collector) will be captured on all data collection forms. Source documents that require handwriting and that are completed by study staff will be filled-out legibly. A signature log will maintained in the regulatory binder, such that the data originator can be easily identified.
3.	Details of all communications with subjects regarding symptoms and study-related events will be documented in questionnaires completed by subjects and in clinical visit flowsheets. A Concomitant Medication Log will be used for subjects participating in clinical trials.
4.	All e-mails that contain any instructions or clarifications regarding study procedures, clinical visits, questions from subjects, information about concomitant medication use, adverse events and health problems, postponed and missed appointments, consultations with study physician, consultations with the principal investigator, and other study-related information will be filed on an ongoing basis in individual subject folders entitled “Subject (ID#) Correspondence.”
5.	Instructions given to subjects either in-person or by e-mail will be transcribed and stored together with other study documents on the university password-protected storage network.
6.	Blank questionnaire items will be reviewed with subjects at the time of study completion to ensure that they were not omitted by mistake. If entries were left blank on purpose, they will be marked with ø symbol, the reviewer initials, and the date of review.
7.	Both Human Subject Protections and Good Clinical Practice training certifications will be on file prior to a staff member's involvement in a clinical trial.
8.	Study-specific Training Logs and meeting minutes will be maintained in the regulatory binder.
9.	Specimen Tracking Logs will be used for the collection, processing, storage, and disposal of all specimens collected from subjects.
10.	Study documents will be updated at the time of each amendment submitted to the IRB.
11.	All pertinent communications with the sponsor will be maintained in the regulatory binder.

*Source documents - All information in original records and certified copies of original records or clinical findings, observations, or other activities in a clinical trial necessary for the reconstruction and evaluation of the trial*.

*Source data - All data contained in source documents (original records or certified copies)*.

## Statistical Analysis Plan

A statistical analysis plan developed *a priori* as part of the planning of the whole RCT is critical to the success of the study (5). The statistical analysis plan has many of the components of the DSMP described in Table 2 for the blueberries and bone RCT; i.e., (1) Study descriptor information, (2) Background and rationale for the study, and (3) Study methods and sample population. It also includes a plan for selecting the sample for final analysis (intent-to-treat, per protocol, completers, safety), testable hypothesis with consideration for the primary and secondary outcomes, and specific approach to be used for statistical analysis. In the blueberry and bone RCT, all data were included for participants who completed at least two out of three intervention periods. No provision for populating the database with missing data or use of covariates were planned. The primary outcome variable, i.e., natural logarithm of urinary ^41^Ca:Ca ratio, was analyzed with a modified general linear model that included terms for participant, time, the participant × time interaction, and the intervention period. This model allows the intervention effects to be estimated from the difference between the intervention period and the non-intervention periods. Exponentiating the differences captures the treatment effect. Standard errors for significance tests were calculated using asymptotic methods and bootstrap procedures. SAS software was used for computations. The statistical model, bootstrap procedure, and sample data are available at http://www.stat.purdue.edu/~mccabe/ca41. A *P*-value was considered significant at <0.05. In our blueberry and bone RCT, no interim analysis was planned. However, advances in clinical trial designs with ability to alter sample sizes and analytical approaches are being explored to conserve resources and minimize subject burden. A special issue of Contemporary Clinical Trials featured examples of innovative and adaptive designs (19).

Best practices for reporting clinical trial progress and results were previously outlined in the paper by Petersen et al. (5). The CONSORT checklist provided in Table 1 covers the fundamental elements of the RCT trial design and statistical analysis plan that need to be reported. A working group convened by the Federation of European Nutrition Societies (FENS) is developing a nutrition extension for the CONSORT checklist to include elements specific to the human nutrition trials (20). Some particular issues that have plagued plant-derived RCTs include not knowing the bioactive constituents or their mechanisms of action, use of biomarkers that do not reflect the health condition of interest, and an enormous placebo effect. In our blueberry RCT, we assumed that the bioactive component responsible for the observed changes in bone turnover were the polyphenols. However, blueberries with different polyphenol profiles have different effects on bone in preclinical studies (9). The bone effect mediated by the endogenous antioxidant and inflammatory pathway was also shown to vary by sex (9). The *in vitro* antioxidant activity of plant compounds does not reflect their physiological activity as previously thought. Thus, both *in vitro* and *in vivo* approaches have been developed to assess the endogenous antioxidant effects (9, 21). Physiological actions could also be mediated by the gut microbiota. Shifts in microbiota suggest that the fiber in the plant material, which serves as a substrate for bacterial metabolism, may also be the bioactive. In a preclinical study of blueberries, a dose-response effect of whole blueberries on the diversity and structure of the gut microbiota was observed; however, there were no significant differences in microbial diversity after feeding blueberry extract (without fiber) (22). Moreover, there were interactions between polyphenol metabolites and shifts in gut microbiota. These potential confounding/mediating factors are important to address in the study protocol and the statistical analysis plan.

Some plant-derived intervention trials have been unsuccessful because of larger than expected placebo effects. This is especially true when the outcome is subjective, as the perception of pain. In a large, multi-center RCT of chondroitin sulfate with and without glucosamine on knee pain, justified by several positive smaller trials, the placebo effect was as large as 60% (23). With such a large placebo effect, it is nearly impossible to quantify the benefit of a bioactive over a comparator.

## Open Data Sharing

The NIH expects researchers and institutions to develop plans for data management and sharing as part of grant applications under a policy effective January 25, 2003 (24). Open sharing of data promotes secondary analyses that advance science and extend the impact of the investment in research including participant efforts. Open data sharing also allows for corrections to the databases thereby increasing the quality of evidence. It is hoped that all funded clinical trial research will adopt open data sharing practices.

Depositing data in a quality data repository generally improves FAIR (findable, accessible, interoperable, and reuseableness) attributes. Multiple data repositories exist for different types of data, with the 20 most frequently mentioned in literature identified by Federer et al. (25). For the blueberry and bone RCT, a National Science Foundation funded platform, Digital Environment for Enabling Data-Driven Science (DEEDS) was used to preserve, document, support, and publish data as online, discoverable datasets (26). To facilitate data sharing and re-use, it would be advantageous to develop one clinical trial repository available to all researchers.

## Implications and Recommendations for Nutrition Research and Policy

Adoption of best practice guidelines for plant-derived interventions in human nutrition RCTs described in this article will increase the rigor of the evidence-base for determining dietary bioactive intake recommendations. Although few countries have attempted to develop dietary guidance for bioactives, a process for using an evidence-based approach for policy makers to establish dietary bioactive intake recommendations based on safety and beneficial health outcomes has recently been published (27). Dietary guidance is only as strong as the strength of the evidence-base. A consistent and transparent evidence-base can facilitate development of robust dietary guidelines for plant-derived compounds, foods, and beverages.

## Conclusions

This paper outlines best practice guidelines for design and conduct of human nutrition RCTs involving plant-derived interventions. These guidelines are intended to promote rigor and transparency of the evidence-base used to establish dietary recommendations for health-promoting plant bioactives. Rigorous and transparent RCTs are needed to allow for causal interpretation of data in diverse populations, across the lifespan, race/ethnicity, and health status variables, and to address the limitations of the current literature for plant bioactives including lack of understanding of the mechanisms, effective and safe doses, and unanticipated effects (28).

## Data Availability Statement

The original contributions presented in the study are included in the article/supplementary material, further inquiries can be directed to the corresponding author.

## Author Contributions

CW conceptualized the topic. CW and JH researched and analyzed the literature and wrote the manuscript including interpretations. All authors approved the final version of the manuscript and agreed to be accountable for all aspects of the work.

## Funding

A fee waiver was provided to CW as a co-editor of the special issue. Many examples used in this article came from a RCT funded by NIH/NICCH grant: R01 AT008754.

## Conflict of Interest

The authors declare that the research was conducted in the absence of any commercial or financial relationships that could be construed as a potential conflict of interest.

## Publisher's Note

All claims expressed in this article are solely those of the authors and do not necessarily represent those of their affiliated organizations, or those of the publisher, the editors and the reviewers. Any product that may be evaluated in this article, or claim that may be made by its manufacturer, is not guaranteed or endorsed by the publisher.
